# Notices, Reviews, &c.

**Published:** 1859-03

**Authors:** 


### NOTICES, REVIEWS, &c.

We have made an arrangement with Hubbard W. Swett, Book and Periodical Dealer, 128 Washington Street, Boston, to act as our Agent in that City, for the following books (our own publications):—Health and Disease, a Book for the People; Consumption, Bronchitis and Kindred Diseases; and the Bound Volumes of the Journal of Health. H. B. S. will supply the trade of Boston at our Lowest Cash prices. We shall establish no agencies for the Monthly Journal, but leave the Sale open to all the Trade out of New York City, can be supplied by the following Wholesale News Agents, Ross & Tousay, 121 Nassau St., Dexter & Bro., 14 and 16 Ann St., and R. M. Dewitt, 160 and 162 Nassau St.

H. B. PRICE, Publisher,
*No. 3 Everett House, New York.*

*The Country Gentleman*, weekly, $2 a year, Albany, New York, is instructive to any family in the city or country, and merits as it receives, a wide and generous patronage.

The *Independent*, New York, $2 a year, has for its special contributors for 1859, John G. Whittier, Harriet Beecher Stow, George B. Chever, Henry Ward Beecher; great names all.

*Ladies American Magazine*, $2 a year, 7 Beekman Street, New York.

"*Opthalmic Hospital;*" New York. Fifth annual report, with an address by Mark Stephenson, M. D. Subject—" Law and Medicine contrasted." Able, scholarly, discriminating.

*Moral Insanity.*—Anything which Dr. Reese may choose to write on an important medical subject, commands the respectful attention of the medical profession. His "report" prepared at the request of the American Medical Association, should be preserved for reference, by every lawyer or physician of any eminence, not only here, but abroad. It is an able and standard contribution to medical jurisprudence.

Millions of eyes have been delighted in past years by the beautiful engravings in "Graham's Magazine," which is now merged in

*The Ladies Magazine*, $2 a year, single numbers 18cts, Henry White, publisher, No. 7 Beekman Street, New York: we cordially wish it the success which it merits.

*The Home*, by Metta Victoria Fuller; $1 50 a year. New York, and Buffalo. Is a safe companion for our wives and daughters.

*Christian Review*, $3 a year; removed from Baltimore to New York, published by Shelden, Blakeman & Co., is one of the very best religious quarterlies in America. Baptist Lutheran Home Journal, $1 50 a year, published monthly at 732 Arch Street, Philadelphia.

*Sixteenth Annual Report* of the Young Men's Social and Benevolent Society of the Presbyterian church and congregation, Fifth Avenue, corner of 19th Street, New York, organized March 22, 1842. The average monthly attendance is two hundred; of the 237 young men who have become members since March 1842, only five have died; this simple statement is one of the strongest proofs that can be given of the life insuring, and life preserving influences of that sobriety of character, of that steadiness and regularity of deportment which belongs to young men in cities whose tastes lead them to a connection with "Christian associations." Let every parent then, from the country, who sends a son to the city to try his fortune, enjoin it upon him, as a means of preserving character, health and life itself, to make it his first business, and by no means to be neglected, to connect himself with one of these "associations," a thousand fold better than any "company" or "club," or "band" in the government.

*American Medical Gazette* for February, edited by Dr. Reese, is the best number issued for variety, ability, and practicality. The world renowned Whitney case—for it is destined to travel through all civilized lands, and to be handed down to posterity, is fully reported. The learned editor comes ably and manfully to the defence of Dr. Horace Greene, who is by common consent, allowed to be one of the best, most honorable, and most eminent medical men at home or abroad. The incontrovertible facts declare, as does every physician in New York, who has taken proper measures for a thorough investigation, that the operator had no agency whatever, directly or indirectly in the death of the patient, and such was our opinion from the first; the very supposition was an absurdity, under the true circumstances of the case.

*Blackwood*, $3, for Feb'y, comes promptly and welcomely.—For sale at our counter.

